# Gestalt, Navon and Kanizsa illusion processing in CVI, ADHD, and dyslexia Children with Normal verbal IQ

**DOI:** 10.3389/fnhum.2024.1496796

**Published:** 2024-12-24

**Authors:** Marinke J. Hokken, Ymie J. Van Der Zee, Rob Rodrigues Pereira, Ingrid G. I. J. G. Rours, Maarten A. Frens, Johannes van der Steen, Johan J. M. Pel, Marlou J. G. Kooiker

**Affiliations:** ^1^Department of Neuroscience, Erasmus Medical Center, Rotterdam, Netherlands; ^2^Royal Dutch Visio, Huizen, Netherlands; ^3^Kinderplein Medical Center, Rotterdam, Netherlands

**Keywords:** cerebral visual impairment (CVI), attention deficit hyperactivity disorder (ADHD), dyslexia, Kanizsa illusory contours, Navon, Gestalt closure, global visual selective attention

## Abstract

**Introduction:**

Global Visual Selective Attention (VSA) is the ability to integrate multiple visual elements of a scene to achieve visual overview. This is essential for navigating crowded environments and recognizing objects or faces. Clinical pediatric research on global VSA deficits primarily focuses on autism spectrum disorder (ASD). However, in children with cerebral visual impairment (CVI) and other neurodevelopmental disorders (ADHD, dyslexia) similar deficits are reported. The aim of this study was to investigate global VSA performance in children with CVI, ADHD, dyslexia and neurotypical children by combing gaze-based measures with conventional neuropsychological tasks.

**Methods:**

We included children aged 6–12 years with CVI (*n* = 20), ADHD (*n* = 30), dyslexia (*n* = 34) and neurotypical development (*n* = 37), all with normal verbal IQ. Eye tracking measurements were stepwise introduced within three global VSA tasks: Gestalt Closure (no eye tracking), Navon stimuli (eye tracking-based qualitative analysis) and Kanizsa Illusory Contours (KIC; eye tracking-based quantitative analysis). Verbal responses were compared with non-verbal gaze behavior.

**Results:**

Children with CVI had significantly lower success rates on Gestalt Closure recognition, prolonged verbal response times on Navon stimuli, and decreased verbal and gaze performance on the KIC task compared to all other groups, irrespective of visual acuity. Children with ADHD and dyslexia performed similar to neurotypical children on all tasks.

**Discussion:**

The results suggest а distinct global VSA deficit in children with CVI, which aligns with clinical observations of symptoms in daily life. Incorporating gaze-based analyses provided new information about search strategies beyond verbal answers and made the KIC task more inclusive for children with language and motor disabilities. Assessing global VSA within clinical CVI assessments could improve the differential diagnostic evaluations among children with CVI, ADHD and dyslexia, leading to more personalized treatment approaches.

## 1 Introduction

The visual world around us contains too many elements to process simultaneously. Hence, visual selective attention (VSA) is crucial to avoid visual overload. VSA acts as a spotlight, focusing on specific parts of the visual field for further processing, while other parts remain outside conscious awareness (Posner et al., 1980). According to theories, the size of this ‘attentional spotlight’ is not fixed, but can be adjusted based on the task at hand. When the attentional spotlight is narrow, known as *local VSA*, individual visual elements are processed in detail. Conversely, when the spotlight is broad, known as *global VSA*, multiple visual elements are processed simultaneously, creating a visual overview that is important for, e.g., segmenting scenes and recognizing objects (Liechty et al., 2003; Förster and Dannenberg, 2010; Förster et al., 2008).

Unlike adults, young children and infants have a local preference as they primarily rely on visual details when exploring a visual scene (Kimchi et al., 2005). The shift to an adult-like global VSA preference typically seems to occur between 5 and 7 years of age (Poirel et al., 2012; Abravanel, 1982; Happé, 1996; Milne and Scope, 2008; Hadad et al., 2010), though some studies suggest it appears earlier (Guy et al., 2013; see for a review Colombo et al., 1991). This developmental shift, however, seems inconsistent in children with neurodevelopmental disorders. These children may retain a local preference, resulting in a fragmented visual processing which hinders their ability to understand larger visual scenes, objects and faces. Lacking a comprehensive view of a visual scene may cause, e.g., difficulties in understanding cause-and-effect relationships and heightened anxiety in crowded scenes such as supermarkets, traffic situations or school yards (Dutton and Jacobson, 2001). Lacking the ability to integrate facial features may lead to misinterpreting social cues and social–emotional difficulties. In children with autism spectrum disorder (ASD), these global VSA deficits have been extensively studied in the context of the weak central coherence theory (Booth and Happé, 2010). Similar daily symptoms have been reported by the parents of children with Cerebral Visual Impairment (CVI), Attention Deficit Hyperacitivy Disorder (ADHD) and dyslexia (Hokken et al., 2024b). Especially in children with CVI, a brain-based visual disorder, global and local VSA deficits are common due to compromised processing within dorsal (i.e., occipital and parietal) cerebral networks (Bennett et al., 2020; Atkinson, 2017). These children often miss important visual details and lack a comprehensive overview over complex scenes, even in the absence of visual acuity or visual field deficits (Atkinson, 2017; Moore and Zirnsak, 2017; Philip and Dutton, 2014; Hokken et al., 2024b). The latter has also been described as simultanagnosia (Chokron and Dutton, 2023). A 12-year-old girl with CVI explained:

‘*I see too many things, too many stimuli around me, and I can’t put it all together quickly. My vision is actually like a puzzle. For example, when I see a face, I first see the mouth, and after that little by little, it becomes a whole*’ (film fragment adapted from Koninklijke Visio, 2019).

**Figure 1:**
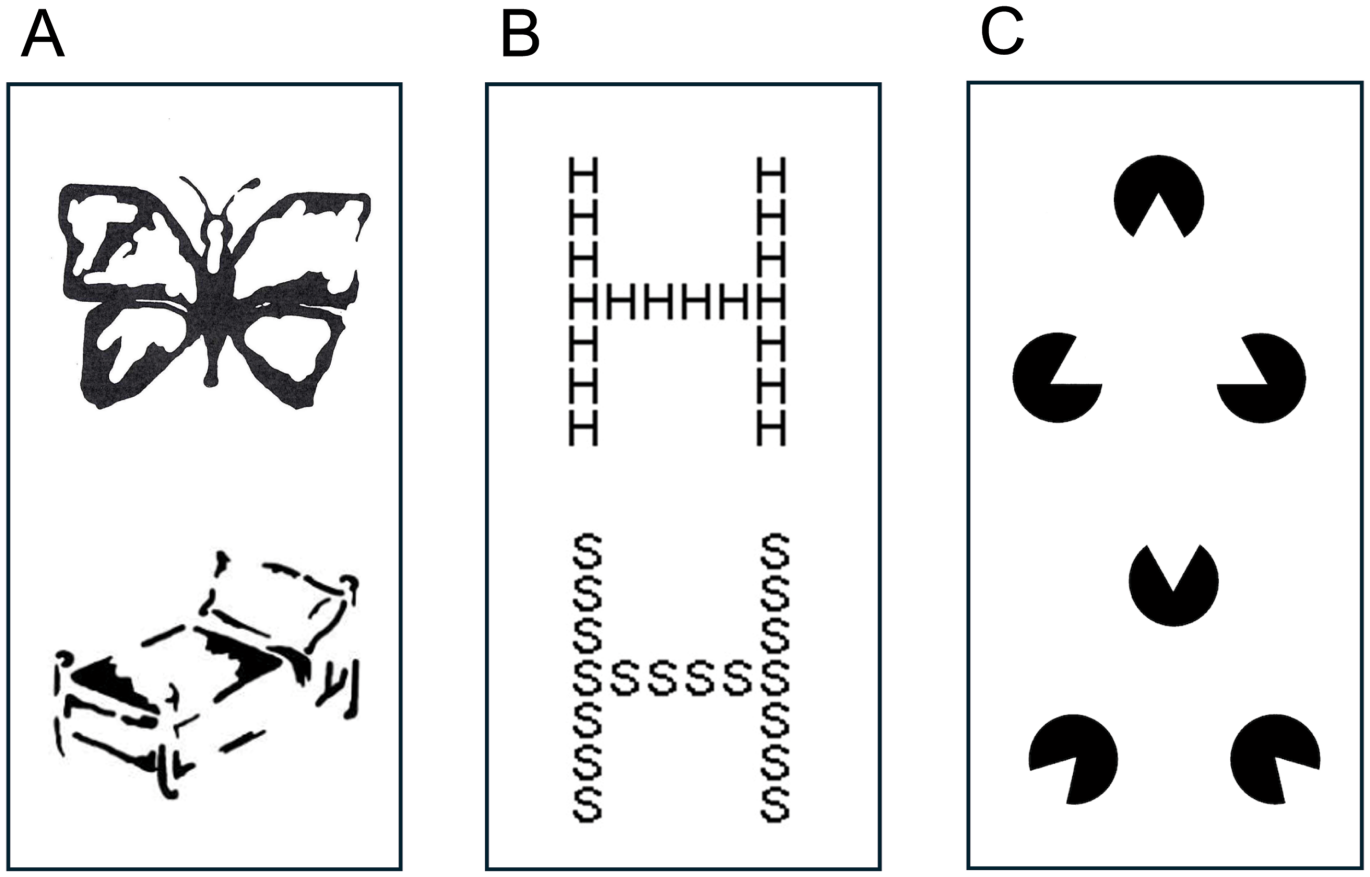
**(A)** Kaufman Gestalt Closure task. **(B)** Navon task: congruent global/local elements (top) and incongruent global/local elements (bottom). **(C)** Kanizsa Illusory Contours: with an illusory contour (top) and without an illusory contour (down).

The neuropsychological assessment of CVI, that focusses on a variety of potential impaired visual functions, often includes tasks designed to evaluate global VSA performance. These tasks typically include a global level that must be perceived independently from the local level of the visual scene or object. In other words, the scene or object cannot be easily identified by solely looking at the details one by one, but only when broadening the attentional spotlight to reach visual overview. Examples of well-known conventional tasks are presented in Figure 1. The Kaufman’s Gestalt Closure task (Figure 1A) consists of incomplete inkblot pictures (Kaufman and Kaufman, 1983). Navon figures (Figure 1B) consist of global large forms, i.e., letters, that are composed of either congruent or incongruent small local elements (Navon, 1977). Kanizsa illusory contours (KICs; Figure 1C) consist of local visual elements (Pac-Man) that are placed in such a way that, in target trials, an illusory shape can be distinguished (Kanizsa, 1976).

Although local VSA deficits have been quite extensively reported and analyzed in children with CVI (Bennett et al., 2018; McKillop and Dutton, 2008; Manley et al., 2022; Manley et al., 2023; Zhang et al., 2022; Hokken et al., 2024a), only a handful of studies investigated global VSA. Weaker performance on global VSA tasks was found in children with CVI (van der Zee et al., 2019), and in children with a medical risk of CVI, such as children with Periventricular leukomalacia (PVL; Rešić et al., 2008; Fazzi et al., 2004) and children born very prematurely (<33 weeks of gestation; Datin-Dorrière et al., 2021). Literature on task performance in ADHD and dyslexia is more inconclusive, as a local before global precedence was found for both children with ADHD (Song and Hakoda, 2012; Song and Hakoda, 2015) and children with dyslexia (Franceschini et al., 2017), but other studies did not (Kalff et al., 2002; McAvinue et al., 2012). Taken together, it is challenging to differentiate between CVI, ADHD, and dyslexia in clinical practice based on global VSA task performance.

Another clinical challenge in assessing global VSA is the limitations of the available, conventional tasks. First, many tests are largely observational and lack a standardized method or comprehensive and updated normative data for children. Second, the tests often require a verbal or hand-motor (e.g., mouse click) component, excluding young children and complicating the disentanglement between potential language (naming), motor deficits (pointing) and visual deficits. Third, these tasks result in binary performance measures (normal-abnormal) without insights into potential deviant VSA processes and strategies during task performance. These issues may be overcome by coupling global VSA tasks with eye tracking and analyzing gaze responses during the tasks. These gaze responses are not only non-verbal and non-motor of nature, a proof-of-concept study in children with CVI (van der Zee et al., 2019) also demonstrated how eye tracking can reveal valuable insights about visual attentional processes. Therefore, it is expected that eye tracking may give more detailed insight in global VSA performance and development over age.

The aim of the present study was to investigate global VSA performance in children with CVI, ADHD, dyslexia and neurotypical children by gradually expanding conventional neuropsychological tasks with gaze-based measures. Eye tracking measurements were stepwise introduced within three global VSA tasks: a conventional Gestalt Closure task (no eye tracking), a verbal Navon task (eye tracking-based qualitative analysis) and the non-verbal Kanizsa Illusory Contours task (KIC; eye tracking-based quantitative analysis). In the latter two tasks, verbal responses were compared with non-verbal gaze behavior. We hypothesized that children with CVI performed weaker on all tasks and showed deviant gaze behavior, e.g., slower responses, compared to all other groups.

## 2 Methods

### 2.1 Participants

This study took place between January 2021 and August 2022, and included children with CVI, ADHD, dyslexia, and neurotypical children between 6 and 12 years of age were included. Children with CVI were recruited via Royal Dutch Visio, a rehabilitation center for blind and visually impaired people. Demographic, ophthalmic, and neurologic data were extracted from the records. Children with ADHD were recruited via MC Kinderplein and AllesKitz (Dutch mental healthcare centers for children). Parents filled out the Dutch ADHD Questionnaire (ADHD Vragenlijst: AVL) to cluster their daily symptoms (Scholte and Van der Ploeg, 2005). Children with dyslexia were recruited via RID (regional institute for dyslexia). All clinical diagnoses were based on official guidelines and confirmed by experienced health care professionals prior to study inclusion. We excluded children with a combination of the above diagnoses from the study. Neurotypical children, serving as the control group, were selected through siblings of participants with CVI and colleagues of Royal Dutch Visio and Erasmus MC. Exclusion criteria were a verbal IQ, or comprehension index (VCI, from WISC-V), below 70, ASD diagnosis, visual acuity below 0.1 decimal (Snellen equivalent) or a visual field less than 30 degrees. Written informed consent was obtained by parents or caregivers before commencing the study. The study was approved by the Medical Ethical Committee of Erasmus MC Rotterdam (MEC-2020-0680) and complied with the tenets of the Declaration of Helsinki (2013) for research involving human subjects.

### 2.2 Procedure and experimental set-up

Children underwent a 45- to 60-min assessment in a noise-free room. The assessment consisted of conventional neuropsychological tasks and novel gaze-based tasks, assessed in a fixed order, with breaks provided between tasks when needed, as part of a larger study into visual selective attention processes (EEVA–Erasmus Eye tracking Visual Assessment). A subset focusing on global VSA was included in the present study.

#### 2.2.1 Conventional neuropsychological tasks

##### 2.2.1.1 WISC-V verbal comprehension index

The child’s verbal intelligence level was measured using the Similarities and Vocabulary subtests from the Dutch adaptation of the Wechsler Intelligence Scale for Children (WISC-V-NL; Wechsler, 2017). The combined score from these subtests was converted into an age-based norm score that determines the Verbal Comprehension Index (VCI).

##### 2.2.1.2 Gestalt closure task

The Gestalt Closure Task is part of the Kaufman Assessment Battery for Children (K-ABC; Kaufman and Kaufman, 1983). The test has been developed for children between three and 18 years of age and has good reliability (*r* = 0.92). The participants were asked to identify 37 incomplete inkblot images of everyday objects and animals printed on paper. When the participant made four consecutive mistakes, the task was ended. Raw scores of the total number of correctly recognized images were converted into age-based norm scores, with a range of 1 to 19.

#### 2.2.2 Gaze-based tasks

Children were placed in front of a 22.5-inch screen (1920 × 1,080 or 1920 × 1,200 resolution) with a viewing distance of approximately 60 cm. A remote eye tracker was attached to the bottom of the screen to record their eye movements (Tobii Pro X3-120). The eye tracker registered the movement of the eyes at 120 Hz and automatically compensated for movements of the head. A standardized five-point calibration was carried out prior to every task. The children were instructed to minimize movement during the tasks. If they moved, the researcher reminded them to remain still and repositioned them correctly. In case of significant movements, the researcher recalibrated the eye-tracker. Gaze data were processed using Tobii Pro Lab Software and Tobii I-VT fixation filter with a velocity threshold of less than 30 degrees per second to classify fixations and higher than 30 degrees per second to classify saccades.

##### 2.2.2.1 Navon number task

**Figure 2:**
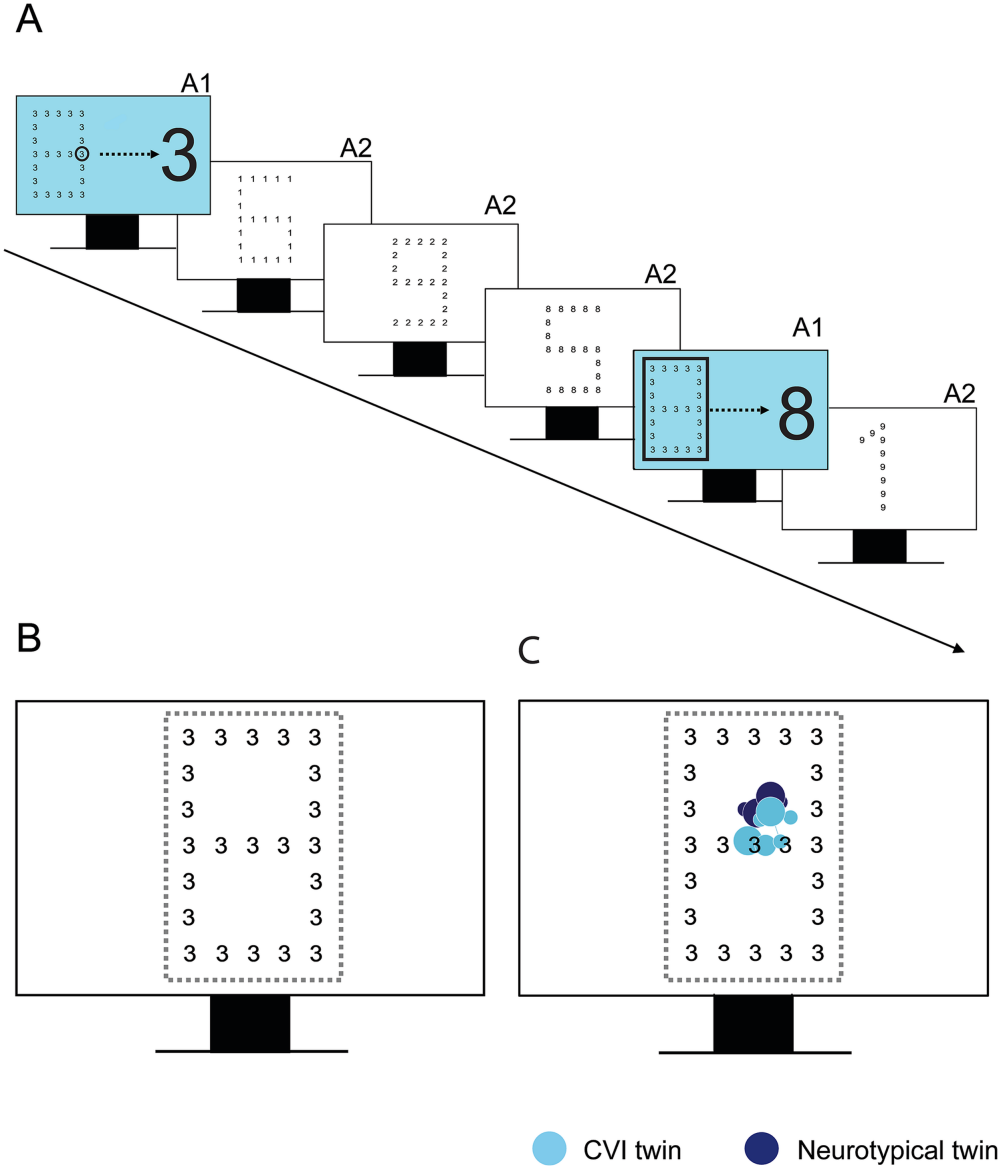
**(A)** Experimental design of the Navon Number Task. For the global and local set, it is explained in practice trials (A1) what level they must report, followed by three test trials (A2). **(B)** Area of interest (AOI) around the Navon stimulus, depicted in yellow. **(C)** Example of gaze behavior in one set of participating twins (12 years of age), one with CVI and one with neurotypical development.

The Navon Number Task (see Figure 2) is a variation on the classic Navon Task (Navon, 1977). Each display showed a large number, i.e., the global level, that was composed of small numbers, i.e., the local level. The task was divided into three sets, all consisting of one practice display and three test displays. In the first set, the participants were given the verbal instruction to name the number that they saw first as quickly as possible (first response set). For the next sets, participants were instructed to name, respectively, the small number (local set) or the large number (global set) as quickly as possible.

Verbal Response Time (VRT) was recorded by the researcher pressing the spacebar directly after the verbal response of the child. The gaze data were used qualitatively, to check the overall attention for the Navon stimuli. We also analyzed the Time to the First Fixation on the AOI, to investigate whether potential delays in VRT were due to slower fixation on the stimulus or processing deficits. Further analysis of saccades and fixations was not included because of the task set up, in which fixation prior to target presentation was not controlled.

##### 2.2.2.2 Kanizsa illusory contours task

**Figure 3:**
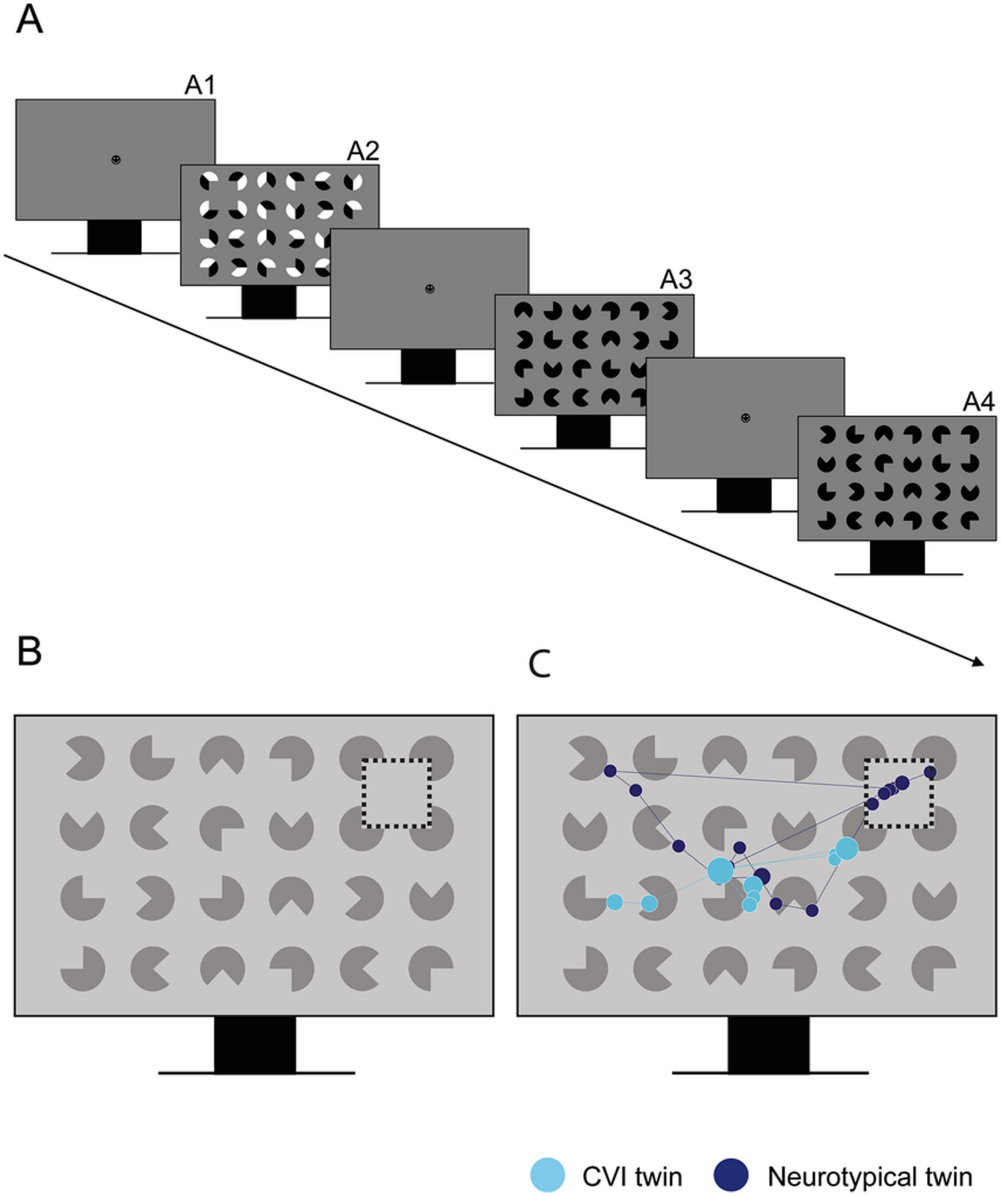
**(A)** Experimental design of the Kanizsa Illusory Contour task. The fixation smiley appears for 1.5 s (A1). The displays consist of black (A3, A4) or black-and-white (A2) Pac-Man stimuli with (A2, A4) or without (A3) an illusory square target. **(B)** Area of interest (AOI) around the Navon stimulus. **(C)** Example of gaze behavior in one set of participating twins (12 years of age), one with CVI and one with neurotypical development.

Figure 3 illustrates the Kanizsa Illusory Contour (KIC) Task, inspired by Kanizsa (Kanizsa, 1976). A total of 10 displays included 48 black or black-and-white three-quarter circles (Pac-Man) stimuli in various rotations. In eight target displays, four Pac-Man were arranged to form an illusory square, positioned in one of the four corners of the display. The other Pac-Man stimuli in the display were placed randomly (no illusory square). There were two non-target trials in which no illusory square was presented. No verbal or motor responses were required during the task. Participants did not receive any explicit verbal instructions, except for the request to look at the screen. After calibration, a fixation smiley first appeared in the middle of the screen for 1.5 s. Then, the Illusory Form displays appeared for 5 s. After all displays were shown, three questions were asked to check whether the child perceived the illusory target: (1) What did you see? (2) What shape did you see? (3) Did you see a square?

For gaze data analyses, an area of interest (AOI) was created surrounding the four Pac-Man forming the illusory square. Outcome measures were Target Accuracy, i.e., whether the child fixated on the AOI, Time to First Fixation on the AOI, and First Fixation Duration on the AOI. In addition, the Visual Search Area, i.e., the percentage of the total display that the child fixated on, was computed by drawing a circle with a radius of 2.5 degree around each detected fixation and marking all pixels within this circle as visited.

### 2.3 Statistical analysis

Statistical analyses were carried out using SPSS Statistics package version 29.0.0. After log-transforming all time-based parameters had a normal distribution. The primary analyses focused on comparing all outcome measures per task between all groups, and on comparing the effect of task conditions within groups. An ANOVA was performed for the Gestalt Closure task. We compared the proportions of correct responses on the KIC Task with a chi-squared test. A MANCOVA was conducted for performance on the Navon Number Task and KIC Task, with age as covariate. Additionally, within-group differences were evaluated on task demands using separate paired sample t-tests per group. For all analysis, the alpha threshold was set at 0.05. Effect sizes were quantified using eta squared, with interpretations categorized as small (*d* = 0.2), medium (*d* = 0.5), or large (*d* = 0.8), following Cohen’s guidelines (1988).

## 3 Results

### 3.1 Group characteristics

In total, 121 school-aged children (6–12 years of age) participated in the study. The groups did not differ in sex (*χ*^2^ = 2.17, *p* = 0.55) and VCI (*F* = 1.07, *p* = 0.36). There was an overall group difference in age (*F* = 3.53, *p* = 0.02, *η*^2^ = 0.08), but post-hoc tests revealed no significant age differences between the separate groups (all *p*’s > 0.05; Table 1).

**Table 1:** Demographic characteristics of the children by group: age, sex and verbal comprehension index of the WISC-V-NL.

	CVI	ADHD	Dyslexia	Neurotypical
N	20	34	30	37
Age	9.5 ± 2.3	10.4 ± 1.4	10.4 ± 1.9	9.4 ± 1.8
Girls (%)	9 (45%)	12 (35%)	16 (53%)	17 (46%)
VCI	96	97	100	104

Within the CVI group, 11 children (55%) were born prematurely (< 36 weeks). Other associated, not mutually exclusive, neurologic findings were pregnancy or birth complications (*n* = 11, 55%), Cerebral Palsy (*n* = 4, 2%), brain structural abnormality (*n* = 4, 2%), epilepsy (n = 4, 2%), opticus glioma (*n* = 1, 5%), Traumatic Brain Injury (*n* = 1, 5%), Hydrocephalus (*n* = 1, 5%), genetic disorder (*n* = 1, 5%). Two children with CVI (10%) had a reduced visual acuity, i.e., between 0.1 and 0.3 decimal. For three children with CVI (24.5%) the visual acuity was suboptimal: between 0.4 and 0.6 decimal. Visual acuity levels were considered in all three tasks. One child (5%) had hemianopia, and one child (5%) had nystagmus. Strabismus was found in seven children (35%), of which two children had exotropia and five had esotropia.

Within the ADHD group, 25 children (74%) had a (sub) clinical score for Attention deficits, 22 children (65%) for Hyperactivity symptoms and 22 children (65%) for Impulsivity symptoms. Thirteen children with ADHD (38%) took medication prior to testing, 21 children did not. Differences between children with ADHD with and without medication were considered for all three tasks.

### 3.2 Gestalt closure

**Figure 4:**
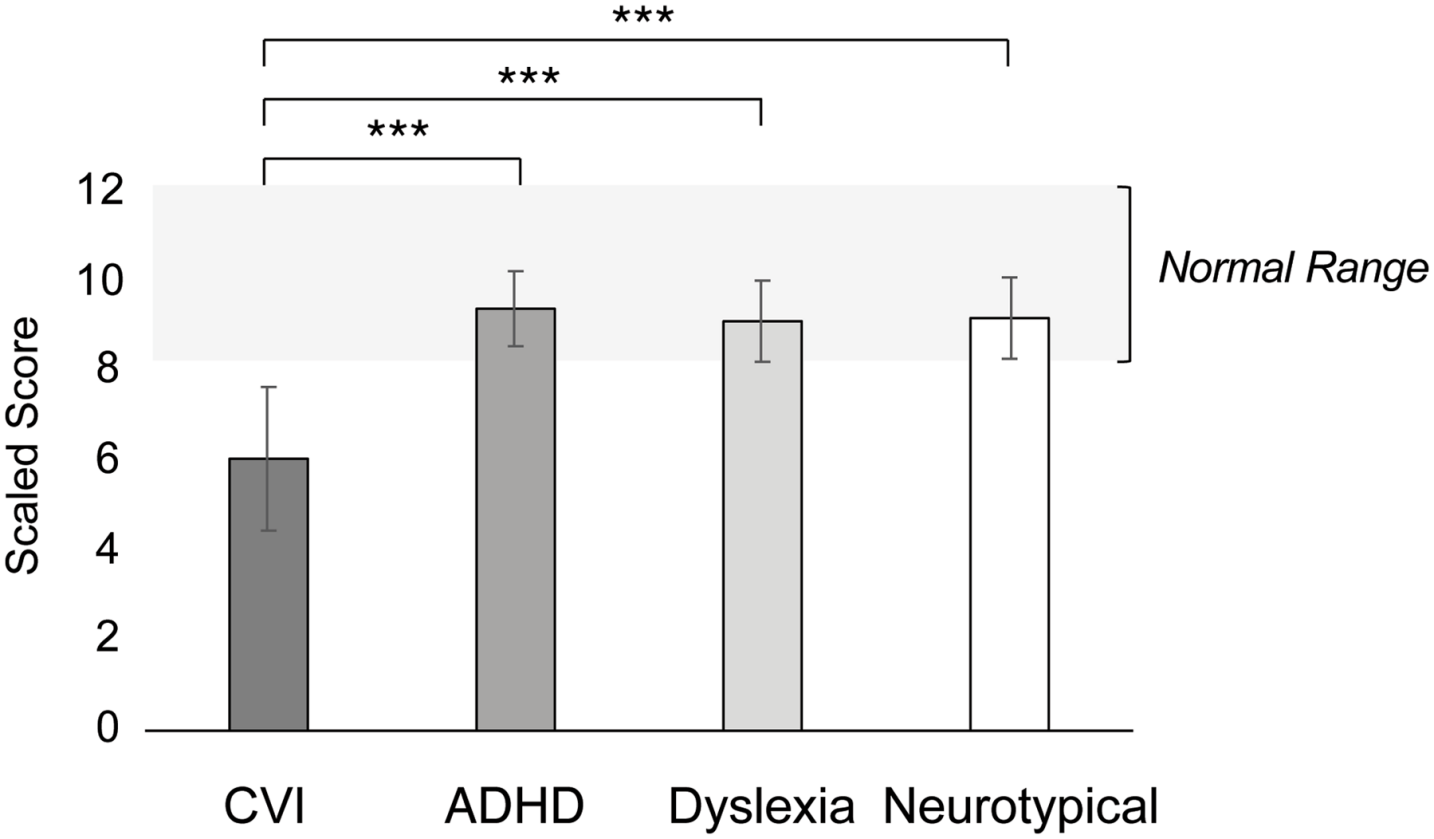
Performance on the Gestalt Closures task, in mean scaled scores with error bars indicating ±1 Standard Error (SE). The light gray bar between the scaled score of 8 and 12 refers to the normal, or average, range. ***p < 0.001, **p < 0.010, *p < 0.05.

Figure 4 shows the Gestalt Closure norm scores per group. Gestalt Closure task performance significantly differed between the groups (*F* = 8.04, *p* < 0.001, *η*^2^ = 0.17). More specific, children with CVI had significantly lower norm scores on the Gestalt Closure task compared to neurotypical children, children with ADHD and children with dyslexia (all *p*’s < 0.001). There was no difference between neurotypical children, children with ADHD and children with dyslexia (all *p*’s = 1.00).

Within the CVI group, there was no correlation between visual acuity and performance on the Gestalt Closure task (*r* = 0.41, *p* = 0.07). Within the ADHD group, medication did not have a significant effect on performance (*F* = 0.00, *p* = 0.99).

### 3.3 Navon number task

**Figure 5:**
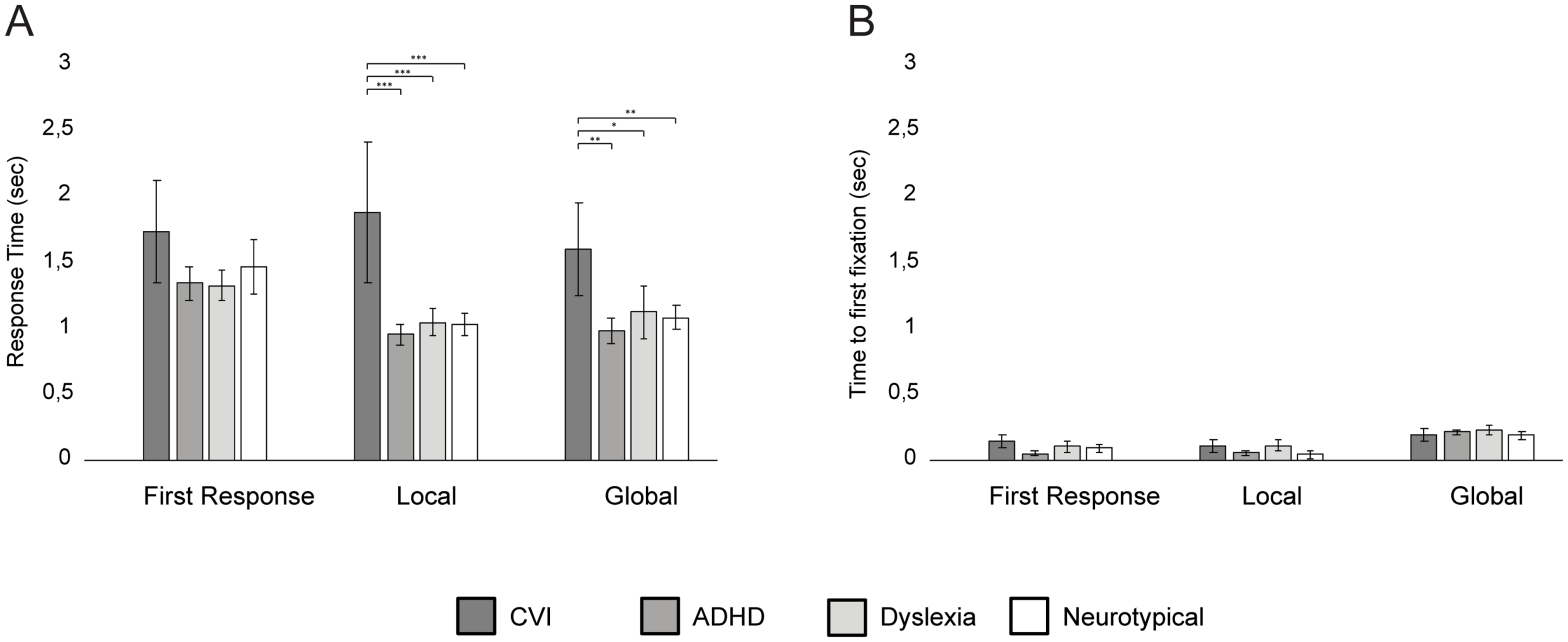
Group differences in mean Verbal Response Times **(A)** and Time to First Fixation **(B)** on the Navon Number Task per set, with error bars indicating ±1 Standard Error (SE). ***p < 0.001, **p < 0.010, *p < 0.05.

In Figure 5, the results of the Navon Number Task are shown per group. Age was a significant covariate for Verbal Response Time (VRT) on the first response set (*F* = 10.58, *p* = 0.005, *η*^2^ = 0.41) and the local set (*F* = 13.74, *p* = 0.002, *η*^2^ = 48), but not on the global set (*F* = 4.41, *p* = 0.05). Age was not a significant covariate for Time to First Fixation for all three sets (all *p*’s > 0.05; Figure 5B).

After controlling for age, there was no group difference in VRT for the first response set (*F* = 1.30, *p* = 0.31), but the groups differed in VRT on the local set (*F* = 23.67, *p* < 0.001, *η*^2^ = 0.83) and the global set (*F* = 7.71, *p* = 0.002, *η*^2^ = 0.61): children with CVI responded significantly slower in the local and global set compared to children with ADHD (local set: *p* < 0.001; global set: *p* = 0.002), children with dyslexia (local set: *p* < 0.001; global set: *p* < 0.016) and neurotypical children (local set: *p* < 0.001, global set: *p =* 0.008).

No differences were found between the other groups (all *p*’s > 0.09; Figure 5A). The Time to First Fixation did not significantly differ between the groups for the first response set (*F* = 0.77, *p* = 0.53), local set (*F* = 2.04, *p* = 0.15) and the global set (*F* = 0.29, *p* = 0.83).

For children with CVI, no correlation was found between visual acuity and VRT on the Navon Number Task (*r* = −0.29, *p* = 0.24). Children with ADHD with or without the use of medication did not differ in performance on the Navon Number Task (all *p*’s > 0.05).

### 3.4 Kanizsa illusory contour task

**Figure 6:**
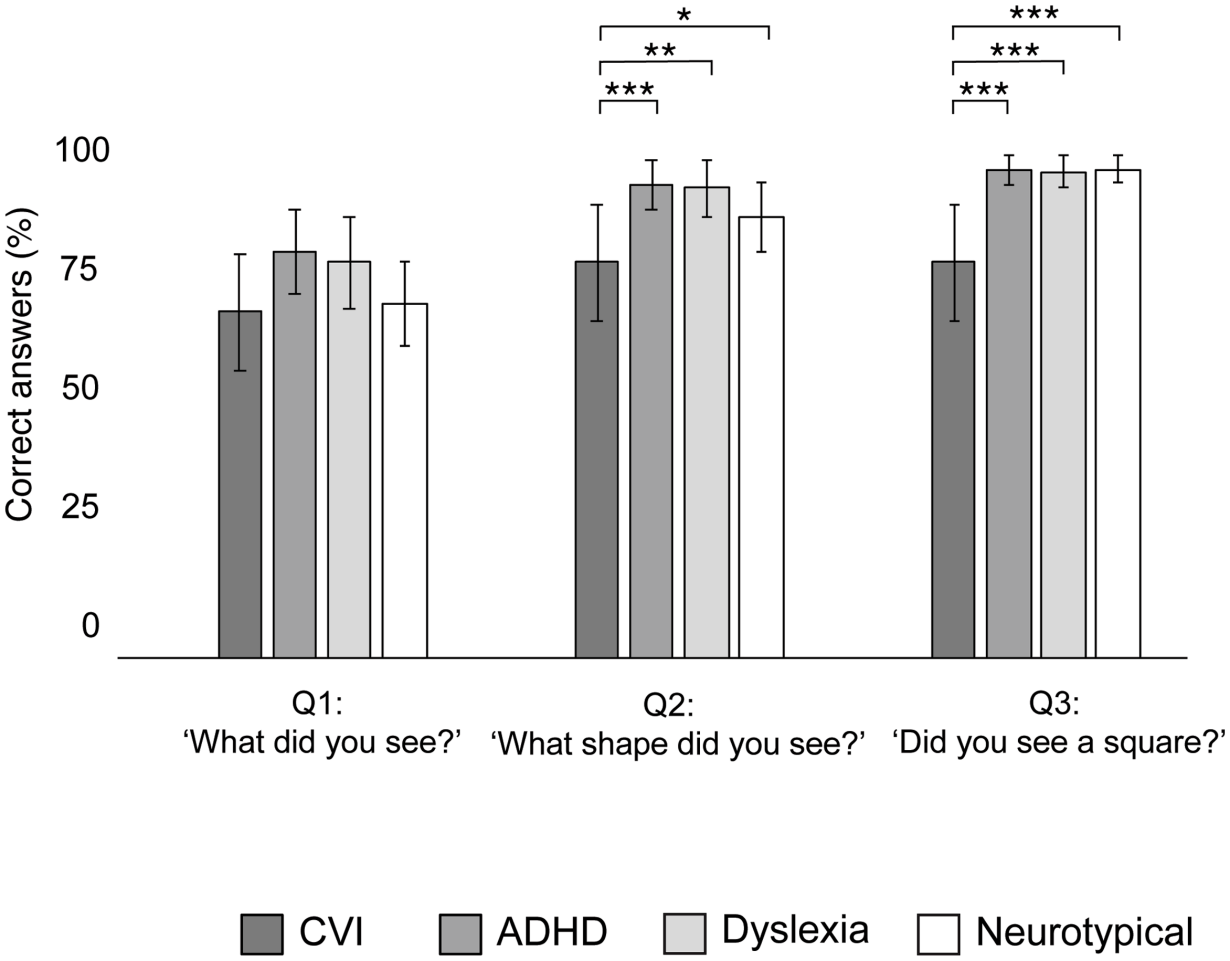
Group proportions of mean correct verbal answers (naming the illusory square) after the Kanizsa Illusory Contour Task, with error bars indicating ±1 Standard Error (SE). ***p < 0.001, **p < 0.010, *p < 0.05.

Figure 6 shows the percentage of correct verbal answers of the children, after the KIC task was completed. These answers did not significant differ between the groups after the first question (‘What did you see?’: *χ*^2^ = 5.23, *p* = 0.16). However, the groups significantly differed in their response on the second question (‘What shape did you see?’: *χ*^2^ = 10.05, *p* = 0.02) and the third question (‘Did you see a square?’: χ^2^ = 27.71, *p* < 0.001). Compared to all other groups, a significant larger proportion of children with CVI did not report the square on question 2 (all *p*’s < 0.05) and 3 (all *p*’s < 0.001). No significant differences in verbal responses were found between the other groups (all *p*’s > 0.05).

**Figure 7:**
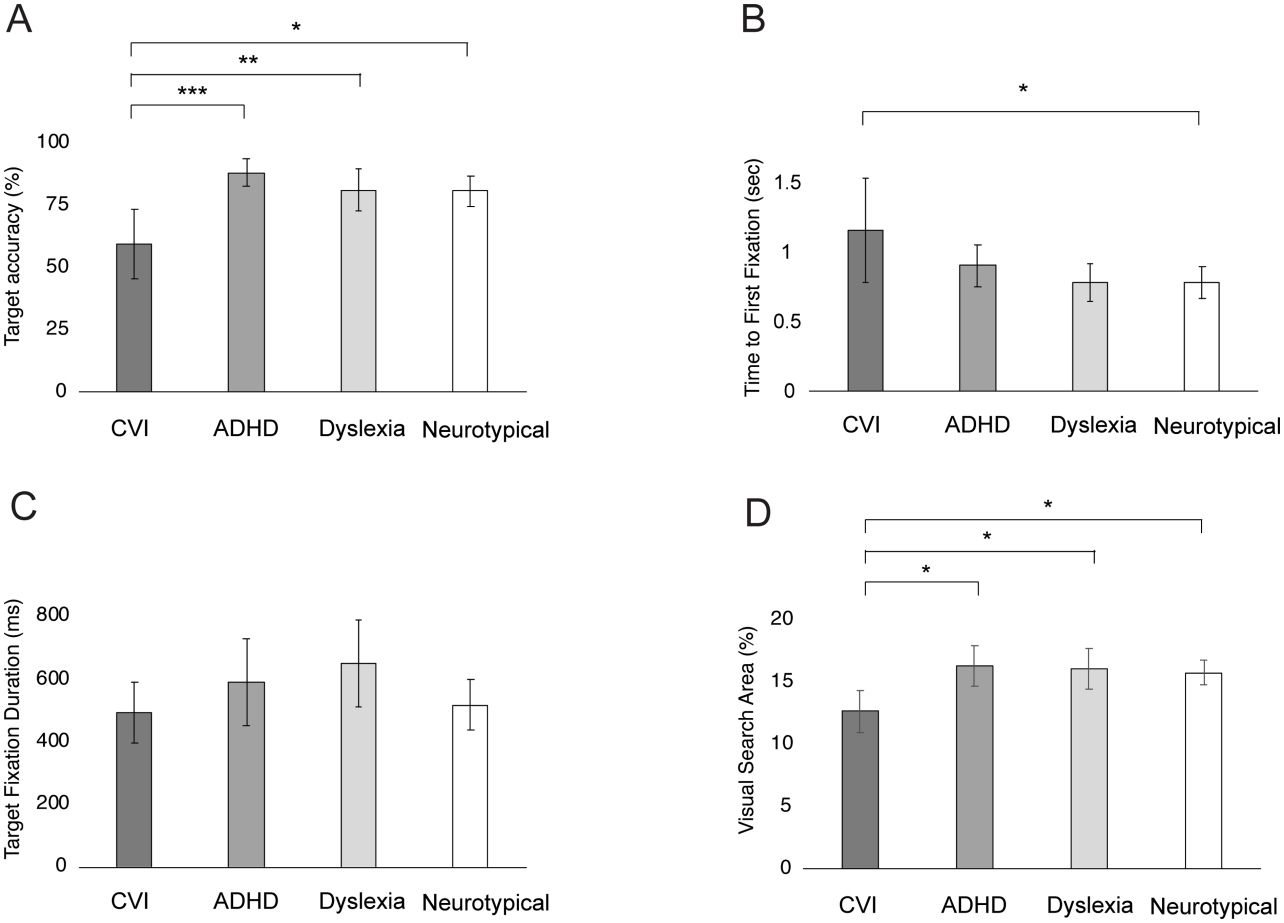
Group differences in gaze behavior on the Kanizsa Illusory Contour Task. Bars represent the mean Target Accuracy **(A)**, Time to First Fixation **(B)**, Target Fixation Duration **(C)**, and Visual Search Area **(D)**, with error bars indicating ±1 Standard Error (SE). ***p < 0.001, **p < 0.010, *p < 0.05.

Figure 7 shows the gaze behavior of the groups on the KIC Task. Age was a significant covariate for Time to First Fixation (*F* = 5.03, *p* = 0.03, *η*^2^ = 0.05), but not for the other parameters (all *p*’s > 0.05).

After controlling for age, a significant group difference was found for Target Accuracy (*F* = 5.72, *p* = 0.006, *η*^2^ = 0.19), Time to First Fixation (*F* = 3.08, *p* = 0.03, *η*^2^ = 0.09) and Visual Search Area (*F* = 3.77, *p* = 0.01, *η*^2^ = 0.10). Children with CVI fixated significantly less often in the target area than children with ADHD (*p* < 0.001), children with dyslexia (*p* = 0.005) and neurotypical children (*p* = 0.01). There was no difference between children with ADHD, dyslexia and neurotypical children (all *p*’s > 0.85; Figure 7A). Children with CVI were significantly slower in the Time to First Fixation than neurotypical children (*p* = 0.04). No differences between the other groups were found (all *p*’s > 0.18; Figure 7B). Children with CVI had a significantly smaller Visual Search Area than children with ADHD (*p* = 0.02), children with dyslexia (*p* = 0.03) and neurotypical children (*p* = 0.02). No differences were found between children with ADHD, dyslexia and neurotypical children (all *p*’s = >0.05; Figure 7D). The groups did not show significant differences in Target Fixation Durations (*F* = 0.59, *p* = 0.62; Figure 7C).

Within the CVI group, there was no correlation between visual acuity and Target Fixations (*r* = −0.181, *p* = 0.446). Within the ADHD group, medication did not have a significant effect on Target Fixations? (*F* = 2.05, *p* = 0.162).

**Figure 8:**
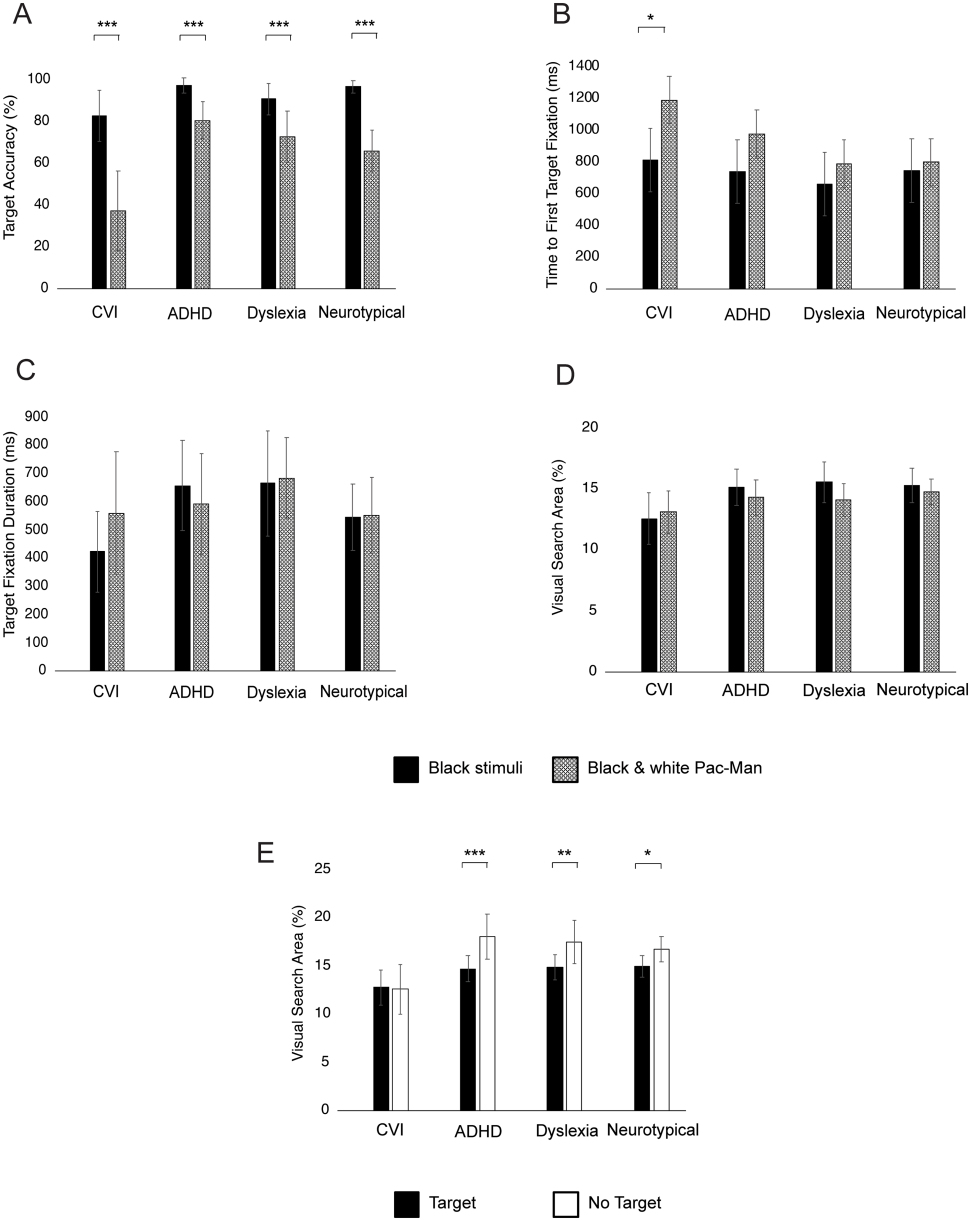
Within-group differences between black and black and white Pac-Mans **(A–D)** and between target and no target conditions **(E)**. Bars represent the mean Target Accuracy **(A)**, Time to First Fixation **(B)**, Target Fixation Duration **(C)**, and Visual Search Area **(D,E)**, with error bars indicating ±1 Standard Error (SE). ***p < 0.001, **p < 0.010, *p < 0.05.

Figure 8 shows the within-group differences between the different KIC conditions. All groups made significantly fewer target fixations (all *p*’s < 0.001) in the black and white condition compared to the black condition (Figure 8A). Children with CVI had significantly faster Time to First Target Fixation in the black conditions compared to the black-and-white condition (*p* = 0.04) but no differences were found within the other groups (all *p*’s > 0.05; Figure 8B). Within all groups there were no differences between black and black-and-white stimuli in Target Fixation Durations (Figure 7C) and Visual Search Area (Figure 8D; all *p*’s > 0.05).

Children with ADHD, dyslexia and neurotypical children had significantly larger Visual Search Areas in the non-target conditions compared to the target conditions (all *p*’s < 0.05). However, in children with CVI there was no difference in Visual Search Area between the target and non-target condition (*p* = 0.87; Figure 8E).

## 4 Discussion

This study examined the global visual selective attention (VSA) deficits that are often reported in the daily lives of children with CVI, but are rarely addressed in scientific research. By comparing children with CVI with their ADHD, dyslexia and neurotypical peers, we found that children with CVI had significant problems with identifying Gestalt Closure pictures, Navon stimuli and Kanizsa Illusory Contours. The gaze-based analyses on two tasks revealed both qualitative and quantitative differences in viewing patterns in children with CVI. In line with previous studies, the impaired performance could not be attributed to visual acuity deficits (Zhang et al., 2022; Manley et al., 2022; Hokken et al., 2024b), suggesting higher order global VSA deficits, potentially due to a dorsal stream vulnerability. More specific, these findings align with the hypothesis that children with CVI have difficulty integrating multiple visual elements into a cohesive whole, which might explain the lack of visual overview they experience in daily life (Chokron and Dutton, 2023; Hokken et al., 2024b).

### 4.1 Global VSA performance and implications for children with CVI

Although significant group differences were found on all three tasks, each task showed unique insights, benefits and limitations for its use in clinical neuropsychological assessments of children with potential global VSA impairments, such as children with CVI.

The results of the Gestalt Closure task, performed on paper, demonstrated discriminative power: Children with CVI consistently performed below the normative, age-based threshold, unlike children with ADHD, dyslexia, or neurotypical development. As was known up front, this task provides limited insight into the underlying processes and strategies beyond the verbal answer. Therefore, it remains unclear whether children with CVI struggled with naming incomplete figures due to a global VSA deficit, or a broader visual identification deficit. Although such isolated visual identification deficits are not commonly reported in children with CVI (Dutton, 2011; Hokken et al., 2024b), and are unlikely given the participants’ average verbal IQ, it is recommended that clinicians compare the results on the Gestalt Closure task with naming tasks that include complete pictures, to ensure correct interpretation of the results.

The results of the Navon Number task, performed on a computer screen with concurrent gaze recordings, showed that children with CVI responded slower to global and local Navon stimuli, but only when the level of analysis (global or local) was prescribed. Qualitative gaze analysis suggested that this delay occurs between stimulus fixation and response, indicating deficits in processing the image or formulating a verbal response. However, further analysis of the cause of this delay, and why this delay is absent when they are free viewing a Navon stimulus, is constrained by the task set-up (see Limitations). Taken together, the Navon Number task requires a refinement of its experimental design before it is a reliable test for clinical practice. Nevertheless, including a Navon task in CVI assessments as an observational tool remains promising, given that it gives valuable insights into whether a child can perceive and switch between local and global levels.

The results of the KIC task, performed on a computer screen with concurrent and detailed gaze recordings, revealed that children with CVI perceived the illusory target less frequently than their peers. Although the first question after the task (‘What did you see?’) might have been too broad to detect group differences, fewer children with CVI recognized or confirmed the presence of a square in their response to the more specific second (‘What shape did you see?’) and third (‘Did you see a square?’) question. These verbal responses align with the measured gaze behavior as they looked less often at the illusory target and were significantly slower in fixating on the black-and-white Pac-Man, indicating greater difficulty with increasing task demands (Bennett et al., 2018; Manley et al., 2022; Zhang et al., 2022; McDowell and Butler, 2023; Hokken et al., 2024a). Drawing from the global VSA theory, children with CVI may perceive the display as a collection of separate elements (Pac-Man elements) rather than a unified illusory shape (Field et al., 1993). As a result, they may have been unaware or uncertain about what to look for and whether it has been found. This could explain the smaller search areas and the lack of expansion of their search when the target was absent. It may also explain the discrepancy with previous studies, which found larger search areas in children with CVI when tasked with finding small details among distractors (Bennett et al., 2018; Manley et al., 2022; Zhang et al., 2022; Hokken et al., 2024a). In those tasks, children with CVI were instructed to search for a specific target but struggled to locate it, causing them to search a larger area of the display. In sum, the KIC Task is promising for clinical CVI assessments, as it has the advantage of enabling analysis of underlying viewing patterns, beyond binary performance outcomes. Additionally, its design, which requires no initial verbal instructions or verbal and motor responses, is particularly inclusive for children with language or motor delays, or for younger children.

### 4.2 Global VSA performance and implications for children with ADHD and dyslexia

Children with ADHD and dyslexia did not differ from their neurotypical peers in verbal and gaze responses across all three tasks, suggesting an absence of global VSA deficits on a group level. This finding contributes to the ongoing debate within the literature. Although a lack of visual overview in everyday settings was reported in children with ADHD and dyslexia compared to neurotypical children, their visual problem scores were significantly lower compared to children with CVI (Hokken et al., 2024b). Thus, whereas VSA deficits are considered a core deficit in children with CVI, this is not the case for children with ADHD or dyslexia. Within ADHD, attentional inhibition, i.e., difficulty in suppressing attention or responses to stimuli, is a core deficit. Attentional inhibition can lead to difficulties in maintaining overview in dynamic environments (American Psychiatric Association, 2013), which can mimic global VSA deficits. Similarly, the core deficit in children with dyslexia lies in phonological processing (Sanfilippo et al., 2020). Reading development generally progresses from a local, letter-by-letter strategy to global whole-word recognition (Ehri, 2005; Perfetti and Hart, 2002). Consequently, the problems children with dyslexia face with text, word and letter overview, could stem from reading delays, rather than global VSA deficits. Therefore, not all children with ADHD and dyslexia may have global VSA deficits, or their deficits are more subtle and not detected by the tasks used in this study. Further research is needed to clarify the presence of global VSA deficits in these groups.

### 4.3 Study strengths and limitations

This study is one of the first to examine the global VSA performance of children with CVI and to compare their verbal and gaze outcomes with those of children with ADHD, dyslexia, and neurotypical development. Given the novelty of this research area, we included multiple short tasks, and stepwise introduced eye tracking-based analysis. As a result, the tasks used were brief, consisted of a fixed number of trials (Navon Number Task and KIC task), and uncontrolled fixation positioning (Navon Number Task). Coupled with relatively small sample sizes, along with the exclusion of children with comorbid diagnoses, these factors may limit the generalizability of our findings. Additionally, the current design of the Gestalt Closure and Navon tasks poses limitations for analyzing VSA through eye movements, as both global and local elements fall within the same areas of interest (AOI), and the shift between the local and global elements is related to a mental shift of attention without physical eye movement, i.e., covert visual attention (Bisley, 2011). Future studies could consider either adjusting the task design to better capture scanning behaviors or exploring alternative measures. For example, pupillometry could provide valuable insights as studies on Navon tasks demonstrated that the pupil constricts more during the selection of local elements compared to the global form (Sabatino DiCriscio et al., 2018; Troiani and DiCriscio, 2017). This might be a first step toward a non-verbal and non-motor assessment of how children perceive Gestalt Closure and Navon stimuli.

Another limitation is that children with ASD were not included in this study. Children with ASD often exhibit global VSA deficits (Booth and Happé, 2010) and they share overlapping daily symptoms with children with CVI. For example, both groups have difficulty with visual overview, avoid crowded and cluttered environments, and experience emotion and/or face recognition deficits (Chokron et al., 2020; Fazzi et al., 2019; Kovarski et al., 2021; Lam et al., 2010; Bauer et al., 2023). However, although children with CVI also benefit from predictable visual environments and structure (McDowell and Budd, 2018; Little and Dutton, 2015), in clinical practice it is often observed that they may be able to compensate by using their verbal and social skills and adapt their behavior to the social norm. Future research should include children with ASD to explore specific differences in gaze behavior compared to children with CVI, especially during global VSA or face recognition tasks.

## 5 Conclusion

In conclusion, this study indicates that VSA deficits in children with CVI involve not only difficulties in locating visual details in crowded environments (local VSA deficits) but also challenges in integrating these details into a unified whole (global VSA deficits). Visual acuity did not affect this global performance, which supports the dorsal vulnerability theory of visual attention deficits in children with CVI. These findings highlight the complexity in higher order visual deficits in children with CVI, both in daily life and in clinical assessments. It further emphasizes the need that tailored CVI interventions address their inability to maintain visual overview.

While further research is necessary, this study offers new insights for improving differential-diagnostic assessments of global VSA in clinical practice. The incorporation of eye tracking in one task provided new information of visual strategies beyond traditional performance measures and enhances the inclusiveness for children with language and/or motor disabilities.

## Data Availability

The datasets presented in this article are not readily available because the authors do not have permission to share data. Requests to access the datasets should be directed to m.hokken@erasmusmc.nl.

## References

[ref1] Abravanel E. (1982). Perceiving subjective contours during early childhood. J. Exp. Child Psychol. 33, 280–287. doi: 10.1016/0022-0965(82)90020-0 7069367

[ref700] American Psychiatric Association (2013). Diagnostic and statistical manual of mental disorders (5th ed.).

[ref3] Atkinson J. (2017). The Davida teller award lecture, 2016: visual brain development: a review of “dorsal stream vulnerability”—motion, mathematics, amblyopia, actions, and attention. J. Vis. 17:26. doi: 10.1167/17.3.26, PMID: 28362900 PMC5381328

[ref4] Bauer C. M. Manley C. E. Ravenscroft J. Cabral H. Dilks D. D. Bex P. J. (2023). Deficits in face recognition and consequent quality-of-life factors in individuals with cerebral visual impairment. Vision 7:9. doi: 10.3390/vision7010009, PMID: 36810313 PMC9944076

[ref5] Bennett C. R. Bailin E. S. Gottlieb T. K. Bauer C. M. Bex P. J. Merabet L. B. (2018). “Assessing visual search performance in ocular compared to cerebral visual impairment using a virtual reality simulation of human dynamic movement” in Proceedings of the technology, mind, and society, 1–6.

[ref6] Bennett C. R. Bauer C. M. Bailin E. S. Merabet L. B. (2020). Neuroplasticity in cerebral visual impairment (CVI): assessing functional vision and the neurophysiological correlates of dorsal stream dysfunction. Neurosci. Biobehav. Rev. 108, 171–181. doi: 10.1016/j.neubiorev.2019.10.011, PMID: 31655075 PMC6949360

[ref7] Bisley J. W. (2011). The neural basis of visual attention. J. Physiol. 589, 49–57. doi: 10.1113/jphysiol.2010.192666 20807786 PMC3039259

[ref8] Booth R. Happé F. (2010). “Hunting with a knife and… fork”: examining central coherence in autism, attention deficit/hyperactivity disorder, and typical development with a linguistic task. J. Exp. Child Psychol. 107, 377–393. doi: 10.1016/j.jecp.2010.06.003, PMID: 20655060 PMC2941847

[ref9] Chokron S. Dutton G. N. (2023). From vision to cognition: potential contributions of cerebral visual impairment to neurodevelopmental disorders. J. Neural Transm. 130, 409–424. doi: 10.1007/s00702-022-02572-8, PMID: 36547695

[ref10] Chokron S. Kovarski K. Zalla T. Dutton G. N. (2020). The inter-relationships between cerebral visual impairment, autism and intellectual disability. Neurosci. Biobehav. Rev. 114, 201–210. doi: 10.1016/j.neubiorev.2020.04.008, PMID: 32298709

[ref11] Colombo J. Mitchell D. W. Coldren J. T. Freeseman L. J. (1991). Individual differences in infant visual attention: are short lookers faster processors or feature processors? Child Dev. 62, 1247–1257. doi: 10.2307/1130804 1786713

[ref12] Datin-Dorrière V. Borst G. Guillois B. Cachia A. Poirel N. (2021). The forest, the trees, and the leaves in preterm children: the impact of prematurity on a visual search task containing three-level hierarchical stimuli. Eur. Child Adolesc. Psychiatry 30, 253–260. doi: 10.1007/s00787-020-01510-x, PMID: 32193647

[ref13] Dutton G. N. (2011). Structured history taking to characterize visual dysfunction and plan optimal habilitation for children with cerebral visual impairment. Dev. Med. Child Neurol. 53:390. doi: 10.1111/j.1469-8749.2010.03900.x, PMID: 21361916

[ref14] Dutton G. N. Jacobson L. (2001). Cerebral visual impairment in children. Semin. Neonatol. 6, 477–485. doi: 10.1053/siny.2001.0078 12014888

[ref15] Ehri L. C. (2005). Learning to read words: theory, findings, and issues. Sci. Stud. Read. 9, 167–188. doi: 10.1207/s1532799xssr0902_4

[ref17] Fazzi E. Bova S. M. Uggetti C. Signorini S. G. Bianchi P. E. Maraucci I.. (2004). Visual–perceptual impairment in children with periventricular leukomalacia. Brain and Development 26, 506–512. doi: 10.1016/j.braindev.2004.02.002, PMID: 15533651

[ref18] Fazzi E. Micheletti S. Galli J. Rossi A. Gitti F. Molinaro A. (2019). “Autism in children with cerebral and peripheral visual impairment: fact or artifact?” in Seminars in pediatric neurology, vol. 31 (Philadelphia: WB Saunders), 57–67. 31548026 10.1016/j.spen.2019.05.008

[ref19] Field D. J. Hayes A. Hess R. F. (1993). Contour integration by the human visual system: evidence for a local “association field”. Vis. Res. 33, 173–193. doi: 10.1016/0042-6989(93)90156-Q, PMID: 8447091

[ref20] Förster J. Dannenberg L. (2010). GLOMOsys: a systems account of global versus local processing. Psychol. Inq. 21, 175–197. doi: 10.1080/1047840X.2010.487849

[ref21] Förster J. Liberman N. Kuschel S. (2008). The effect of global versus local processing styles on assimilation versus contrast in social judgment. J. Pers. Soc. Psychol. 94, 579–599. doi: 10.1037/0022-3514.94.4.579, PMID: 18361673

[ref22] Franceschini S. Bertoni S. Gianesini T. Gori S. Facoetti A. (2017). A different vision of dyslexia: local precedence on global perception. Sci. Rep. 7:17462. doi: 10.1038/s41598-017-17626-1, PMID: 29234050 PMC5727118

[ref24] Guy M. W. Reynolds G. D. Zhang D. (2013). Visual attention to global and local stimulus properties in 6-month-old infants: individual differences and event-related potentials. Child Dev. 84, 1392–1406. doi: 10.1111/cdev.12053, PMID: 23379931

[ref25] Hadad B. S. Maurer D. Lewis T. L. (2010). The development of contour interpolation: evidence from subjective contours. J. Exp. Child Psychol. 106, 163–176. doi: 10.1016/j.jecp.2010.02.003, PMID: 20227089

[ref26] Happé F. G. (1996). Studying weak central coherence at low levels: children with autism do not succumb to visual illusions: a research note. J. Child Psychol. Psychiatry 37, 873–877. doi: 10.1111/j.1469-7610.1996.tb01483.x, PMID: 8923230

[ref27] Hokken M. J. Stein N. Pereira R. R. Rours I. G. Frens M. A. van der Steen J.. (2024a). Eyes on CVI: eye movements unveil distinct visual search patterns in cerebral visual impairment compared to ADHD, dyslexia, and neurotypical children. Res. Dev. Disabil. 151:104767. doi: 10.1016/j.ridd.2024.104767, PMID: 38861794

[ref28] Hokken M. J. van der Zee Y. J. van der Geest J. N. Kooiker M. J. (2024b). Parent-reported problems in children with cerebral visual impairment: improving the discriminative ability from ADHD and dyslexia using screening inventories. Neuropsychol. Rehabil., 1–21. doi: 10.1080/09602011.2024.2328875, PMID: 38502713

[ref29] Kalff A. C. Hendriksen J. G. M. Kroes M. Vles J. S. H. Steyaert J. Feron F. J. M.. (2002). Neurocognitive performance of 5-and 6-year-old children who met criteria for attention deficit/hyperactivity disorder at 18 months follow-up: results from a prospective population study. J. Abnorm. Child Psychol. 30, 589–598. doi: 10.1023/A:1020859629994, PMID: 12481973

[ref30] Kanizsa G. (1976). Subjective contours. Sci. Am. 234, 48–52. doi: 10.1038/scientificamerican0476-48 1257734

[ref31] Kaufman A. S. Kaufman N. L. (1983). Kaufman assessment battery for children. Psychol. Assess. 1, 205–218. doi: 10.1177/073428298300100301

[ref33] Kimchi R. Hadad B. Behrmann M. Palmer S. E. (2005). Microgenesis and ontogenesis of perceptual organization: evidence from global and local processing of hierarchical patterns. Psychol. Sci. 16, 282–290. doi: 10.1111/j.0956-7976.2005.01529.x, PMID: 15828975

[ref34] Koninklijke Visio. (2019). What is CVI? The story of Christy, Floris, and Bertine (English version) [video]. YouTube. Available at: https://youtu.be/9wvGZiTDwa8?si=lWRCYo13Q1yrFSno (Accessed September 5, 2024)

[ref36] Kovarski K. Caetta F. Mermillod M. Peyrin C. Perez C. Granjon L.. (2021). Emotional face recognition in autism and in cerebral visual impairments: in search for specificity. J. Neuropsychol. 15, 235–252. doi: 10.1111/jnp.12221, PMID: 32920927

[ref37] Lam F. C. Lovett F. Dutton G. N. (2010). Cerebral visual impairment in children: a longitudinal case study of functional outcomes beyond the visual acuities. J. Visual Impairment & Blindness 104, 625–635. doi: 10.1177/0145482X1010401008

[ref38] Liechty J. Pieters R. Wedel M. (2003). Global and local covert visual attention: evidence from a bayesian hidden markov model. Psychometrika 68, 519–541. doi: 10.1007/BF02295608

[ref39] Little S. Dutton G. N. (2015). Some children with multiple disabilities and cerebral visual impairment can engage when enclosed by a 'tent': is this due to Balint syndrome? Br. J. Vis. Impair. 33, 66–73. doi: 10.1177/0264619614553860

[ref40] Manley C. E. Bauer C. M. Bex P. J. Merabet L. B. (2023). Impaired visuospatial processing in cerebral visual impairment revealed by performance on a conjunction visual search task. Br. J. Vis. Impair. 41, 214–224. doi: 10.1177/02646196231187550 PMC11756917 39850325

[ref41] Manley C. E. Bennett C. R. Merabet L. B. (2022). Assessing higher-order visual processing in cerebral visual impairment using naturalistic virtual-reality-based visual search tasks. Child. Aust. 9:1114. doi: 10.3390/children9081114, PMID: 35892617 PMC9331719

[ref42] McAvinue L. P. Vangkilde S. Johnson K. A. Habekost T. Kyllingsbæk S. Bundesen C.. (2012). A componential analysis of visual attention in children with ADHD. J. Atten. Disord. 19, 882–894. doi: 10.1177/1087054712461935, PMID: 23190613

[ref43] McDowell N. Budd J. (2018). The perspectives of teachers and paraeducators on the relationship between classroom clutter and learning experiences for students with cerebral visual impairment. J. Visual Impairment & Blindness 112, 248–260. doi: 10.1177/0145482X1811200304

[ref44] McDowell N. Butler P. (2023). Validation of the Austin assessment: a screening tool for cerebral visual impairment related visual issues. PLoS One 18:e0293904. doi: 10.1371/journal.pone.0293904, PMID: 37917596 PMC10621811

[ref45] McKillop E. Dutton G. N. (2008). Impairment of vision in children due to damage to the brain: a practical approach. British and Irish Orthoptic J. 5, 45–50. doi: 10.22599/bioj.222

[ref46] Milne E. Scope A. (2008). Are children with autistic spectrum disorders susceptible to contour illusions? Br. J. Dev. Psychol. 26, 91–102. doi: 10.1348/026151007X202509

[ref47] Moore T. Zirnsak M. (2017). Neural mechanisms of selective visual attention. Annu. Rev. Psychol. 68, 47–72. doi: 10.1146/annurev-psych-122414-033400 28051934

[ref600] Navon D. (1977). Forest before trees: the precedence of global features in visual perception. Cognitive Psychol. 9, 353–383.

[ref49] Perfetti C. A. Hart L. (2002). “The lexical quality hypothesis” in Precursors of functional literacy. eds. Verhoeven L. Elbro C. Reitsma P. (Pittsburgh: John Benjamins Publishing), 67–86.

[ref50] Philip S. S. Dutton G. N. (2014). Identifying and characterising cerebral visual impairment in children: a review. Clin. Exp. Optom. 97, 196–208. doi: 10.1111/cxo.12155 24766507

[ref51] Poirel N. Cassotti M. Beaucousin V. Pineau A. Houdé O. (2012). Pleasant emotional induction broadens the visual world of young children. Cognit. Emot. 26, 186–191. doi: 10.1080/02699931.2011.589430, PMID: 21824012

[ref52] Posner M. I. Snyder C. R. R. Davidson B. J. (1980). Attention and the detection of signals. J. Exp. Psychol. Gen. 109, 160–174. doi: 10.1037/0096-3445.109.2.160 7381367

[ref53] Rešić B. Tomasović M. Kuzmanić-Šamija R. Lozić M. Rešić J. Solak M. (2008). Neurodevelopmental outcome in children with periventricular leukomalacia. Coll. Antropol. 32, 143–147. 18405074

[ref54] Sabatino DiCriscio A. Hu Y. Troiani V. (2018). Task-induced pupil response and visual perception in adults. PLoS One 13:e0209556. doi: 10.1371/journal.pone.0209556, PMID: 30586398 PMC6306195

[ref55] Sanfilippo J. Ness M. Petscher Y. Rappaport L. Zuckerman B. Gaab N. (2020). Reintroducing dyslexia: early identification and implications for pediatric practice. Pediatrics 146. doi: 10.1542/peds.2019-3046, PMID: 32576595 PMC7329249

[ref56] Scholte E. M. Van der Ploeg J. D. (2005). Handleiding adhd-vragenlijst (avl). Houten: Bohn Stafleu Van Loghum.

[ref58] Song Y. Hakoda Y. (2012). The interference of local over global information processing in children with attention deficit hyperactivity disorder of the inattentive type. Brain and Development 34, 308–317. doi: 10.1016/j.braindev.2011.07.010, PMID: 21862271

[ref59] Song Y. Hakoda Y. (2015). Lack of global precedence and global-to-local interference without local processing deficit: a robust finding in children with attention-deficit/hyperactivity disorder under different visual angles of the Navon task. Neuropsychology 29, 888–894. doi: 10.1037/neu0000213, PMID: 26146856

[ref62] Troiani V. DiCriscio A. (2017). Superior abilities to focus visual attention and pupil dynamics are linked with broader autism traits. J. Vis. 17:636. doi: 10.1167/17.10.636

[ref64] Van der Zee Y. J. Kooiker M. J. Talamante Ojeda M. Pel J. J. (2019). Gestalt perception in children with visual impairments: item-specific performance and looking behavior. Dev. Neuropsychol. 44, 296–309. doi: 10.1080/87565641.2019.1590836, PMID: 30880487

[ref65] Wechsler D. (2017). Wechsler intelligence scale for children – Nederlandstalige bewerking [Wechsler intelligence scale for children – Dutch edit]. 5th. Edn: Pearson Benelux B.V.

[ref67] Zhang X. Manley C. E. Micheletti S. Tesic I. Bennett C. R. Fazzi E. M.. (2022). Assessing visuospatial processing in cerebral visual impairment using a novel and naturalistic static visual search task. Res. Dev. Disabil. 131:104364. doi: 10.1016/j.ridd.2022.104364, PMID: 36334401 PMC10331636

